# Sensory Processing in People Experiencing Homelessness in Spain: A Pilot Study

**DOI:** 10.3390/healthcare13243316

**Published:** 2025-12-18

**Authors:** Alicia Cal-Herrera, Berta Gándara-Gafo, Ariadna Corbella-González, Pablo A. Cantero-Garlito, Sonia Panadero-Herrero, Olga I. Fernández-Rodríguez, Begoña Polonio-López

**Affiliations:** 1 INPRO (Research in Occupational Participation and Performance), Department of Health Sciences, European University Miguel de Cervantes, 47012 Valladolid, Spain; alcal@uemc.es (A.C.-H.); acorbella@uemc.es (A.C.-G.); oifernandez@uemc.es (O.I.F.-R.); 2 Promotion Research Unit (INTEGRA SAÚDE), Department of Health Sciences, Health Integration, Faculty of Health Sciences, University of A Coruña, 15006 A Coruna, Spain; berta.gandara.gafo@udc.es; 3 Facultad de Ciencias de la Salud, University of de Castilla-La Mancha, 45600 Talavera de la Reina, Spain; 4 Department Clinical Psychology, Universidad Complutense de Madrid, 28040 Madrid, Spain; spanadero@psi.ucm.es; 5 Technological Innovation Applied to Health Research Group (ITAS Group), Department of Nursing, Physiotherapy and Occupational Therapy, University of de Castilla-La Mancha, 45600 Talavera de la Reina, Spain; begona.polonio@uclm.es

**Keywords:** occupational therapy, homelessness, sensory profile, sensory reactivity, sensory modulation, mental health

## Abstract

Introduction: People experiencing homelessness (PEH) often live in adverse and changing environments and have high rates of mental illness and social exclusion, factors that could influence information processing. However, it is unknown whether these conditions could be related to sensory processing problems. Objectives: Analyse sensory processing in PEH. Methodology: A descriptive cross-sectional study was conducted with 150 participants (mean age of 47.43 ± 10.94 years), using the Adult/Adolescent Sensory Profile and a sociodemographic questionnaire. Results. PEH aged 18–64 showed significantly higher scores in low registration (M = 36.9), sensory sensitivity (M = 41.1) and sensation avoiding (M = 45.5) compared to the control group (*p* < 0.001), suggesting a distinct form of sensory processing in this population. Discussion: These results may be linked to factors such as chronic exposure to unpredictable and stressful situations, as well as to the presence of diverse mental health issues. Conclusion: It is important to consider the sensory characteristics of this population when designing person-centered interventions, in order to reduce social isolation and promote self-regulation strategies, environmental adaptation and greater occupational participation.

## 1. Introduction

The increase in people experiencing homelessness (PEH) represents one of the greatest social challenges worldwide. It is estimated that approximately 150 million people around the world are currently affected by this situation [1], including 895,000 individuals in Europe [2] and an incidence rate of 86.6 cases per 100,000 inhabitants in Spain [3].

The European Federation of National Organisations Working with the Homeless (FEANTSA) defines PEH as individuals who are unable to obtain or maintain adequate housing suited to their needs due to economic and social factors, leading to a situation of social exclusion [4]. This condition entails physical, social, and emotional deprivation, encompassing circumstances ranging from street homelessness to temporary accommodation or inadequate housing [5]. To enhance understanding of this phenomenon, FEANTSA developed the European Typology of Homelessness and Housing Exclusion (ETHOS) [5], which conceptualizes a home based on three dimensions: physical (adequate space for living), social (capacity to maintain meaningful social relationships) and legal (security of tenure) [6,7]. The absence of one or more of these dimensions indicates homelessness or housing exclusion [8], providing a comprehensive framework for analysing and addressing the diverse forms of housing deprivation. Moreover, within the ETHOS framework, homelessness is also conceptualized as a broad context of instability that is shaped by living within institutional regulations that limit their day-to-day life.

Homelessness is a multicausal phenomenon resulting from the convergence of structural and individual factors. At the structural level, key contributors include poverty, unemployment, restrictive housing access policies and limited access to social and health protection systems. At the individual level, experiences of violence, abuse, physical illness and mental health problems play a significant role [9,10,11,12,13,14,15,16]. According to the most recent data available in Spain, 28,552 people experiencing homelessness were assisted in care centres located in municipalities with more than 20,000 inhabitants, with a higher prevalence among men and a mean age of 43 years [3]. Regarding mental health problems, the most frequent disorders include personality disorders, schizophrenia, bipolar disorder, depression, and substance use or abuse [17,18]. Specifically concerning depression, data indicate that 59.6% of PEH present depressive symptoms, with a higher incidence among women; moreover, 40.5% of this group report light or moderate substance use [19].

Concerning the general population, mental health problems have been associated with difficulties in sensory processing, such as sensory reactivity [20,21,22,23,24,25]. Sensory processing is defined as the recognition, organization, and interpretation of sensory information [26]. Meanwhile sensory reactivity is characterized by difficulties in regulating responses to incoming sensory stimuli to which most people readily adapt [27], leading to maladaptive behaviours that impact participation and occupational performance. This impact on occupational participation may interfere with recovery processes and contribute to social isolation [28,29,30,31,32], something that has not yet been studied with PEH.

From this standpoint, ETHOS profiles can be understood as markers of varying forms of residential exclusion that, when considered together with sensory processing, provides a theoretical basis for examining how PEH process stimuli in unstable and unpredictable settings.

To understand how homeless individuals process information, it is helpful to first consider existing studies in other populations, where several authors have developed theoretical models to explain sensory reactivity and related difficulties. Dunn’s Model [33], identifies four different sensory profiles based on two dimensions: the neurological threshold (high or low) and self-regulation strategies (active or passive) in response to sensory stimuli. A low threshold indicates that the individual requires only a low intensity or small amount of stimulation to elicit a reaction, whereas a high threshold means that strong or intense stimuli are needed [33,34]. The neurological threshold is necessary to understand behavioural responses to various sensory inputs, which may be modulated by self-regulation strategies. Dunn’s Model distinguishes four sensory patterns: low registration, sensation seeking, sensory sensitivity, and sensation avoiding [33]. Individuals with low registration exhibit difficulty noticing stimuli of typical intensity or frequency and often fail to detect them, showing delayed or absent responses. This pattern has been associated with conformist behaviours, lower self-esteem, higher levels of stress and anxiety, and difficulties in occupational performance [35,36]. In contrast, sensation seeking individuals actively pursue sensory experiences to compensate for a high neurological threshold. Such behaviours may manifest as excessive affection or frequent attempts to initiate physical contact, which in some cases can be perceived as risky or inappropriate behaviours. A pattern that has been linked to trait characteristic of extraversion [36]. People with sensory sensitivity are characterized by early detection of sensory stimuli due to a low neurological threshold, combined with passive self-regulation strategies; a profile that has been associated with increased shyness, intolerance, and social discomfort [37]. Conversely, sensation avoiding individuals also present a low neurological threshold but employ active self-regulation strategies, engaging in avoidance behaviours to limit exposure to sensory input [33]. This pattern has been linked to aggressive and negative reactions to stimuli perceived as intense or overwhelming [26]. Negative reactions may be expressed as social withdrawal and avoidance of activities [35,36].

Based on Dunn’s Model, various sensory questionnaires have been developed for different age groups, such as the Adolescent/Adult Sensory Profile (AASP) [33], designed for individuals aged 11 years and older. This questionnaire has been culturally adapted and validated for the Spanish population [38,39] and has been used in several studies to examine differences in sensory processing among individuals with mental health disorders, including psychotic disorders, mood disorders, bipolar disorder, post-traumatic stress disorder, and obsessive–compulsive disorder, across different countries [40,41,42,43,44,45,46].

Beyond the absence of housing, homelessness is associated with a higher risk of developing physical or mental health conditions that could contribute to increased social exclusion [47]. Mental health problems are known to negatively affect mood and behaviour, interfere with daily functioning and generate increased emotional challenges that might, in turn, act as triggers for homelessness [17]. Additional intrinsic factors that are common to PEH such as ongoing deprivation and continuous exposure to hostile environments could also increase vulnerability and stress [23,48,49], limit participation, and perpetuate the cycle of homelessness [50,51], which makes it important to understand how homeless people process information to better tackle this issues.

Although there is extensive research on poverty and social exclusion, studies addressing sensory processing difficulties in this population are virtually non-existent and could help understand this phenomenon. Further exploration of this area might provide new theoretical and practical insights and guide the design of personalized interventions aimed at improving the quality of life and social inclusion of PEH.

Therefore, the aim of this study was to analyse and describe sensory processing patterns in a sample of PEH. More specifically, we sought to answer the following research questions:

- Compared with the normative values found in the general population, do PEH show differences in sensory processing patterns?
- Among PEH, are there differences in sensory processing between those with a mental health disorder and those without?

## 2. Materials and Methods

### 2.1. Design

This study had a cross-sectional descriptive design [52], and was conducted in Spain following the STROBE guidelines [53]. Participant recruitment was carried out in collaboration with two organizations that offer services to PEH in the city of Valladolid (Castilla y León, Spain). A protocol was developed which involved the principal investigator’s presence (first author) at the centres in order to recruit participants and collect data between April and December 2023.

### 2.2. Sample Size and Strategy

In order to determine the sample size, data from the most recent 2022 national survey of centers and services for people experiencing homeless in Spain were used as a reference, which indicates a total of 28,552 PEH [3]. Considering that Valladolid is a medium-sized city, it was estimated that approximately 448 individuals might be experiencing homelessness. Based on this estimation, it was determined that the minimum sample size for this study was 140 participants. The statistical analysis was calculated using EPIDAT Version 3.1 [54] with a 95% confidence level, a 3% margin of error and a 10% replacement rate.

Data collection among PEH presents significant methodological challenges due to the unstable and changing nature of their living conditions. This population is often characterized by high geographic mobility and limited permanence in a single location, which complicates their identification and follow-up. Moreover, distrust toward institutions and researchers represents an additional barrier to participation. Given the difficulty of accessing the target population and the high likelihood of missed scheduled appointments, a convenience sampling approach was adopted. In order to address the issues this approach might present, the principal investigator implemented several strategies to maximize the sample that was initially reached regarding their participation in the study, including visiting different centres at various times of day.

A total of 162 PEH were invited to participate, of whom 150 agreed to take part in the study, resulting in a participation rate of 92.59%.

### 2.3. Participants

The sample comprised 150 PEH assisted by two organizations that offer services to homeless people. Eligible participants included individuals who met criteria 1–8 of the ETHOS scale (Appendix A), that were between 18 and 80 years of age, and were able to understand the Spanish language. Individuals presenting moderate or severe cognitive impairment, as well as those under international protection, were excluded from the study.

### 2.4. Procedures

The assessments were conducted in shelters, soup kitchens and day centers for PEH, as well as on the streets. Data were collected by the principal investigator through face-to-face interviews carried out using a printed questionnaire dossier. Once participants provided informed consent, the questionnaires were administered.

During the data collection phase, interviews were conducted in spaces previously selected for their suitability, ensuring privacy and minimizing distracting stimuli. Participants were also given the option to choose the location, date, and time they considered most convenient, in order to promote their comfort and willingness to participate.

Regarding duration and data completion of questionnaires, the interviews did not follow a standardized time frame since they were adapted to the individual needs and pace of each participant, and the principal investigator ensured that all of the items of the evaluation tools were completed, which resulted in the absence of lost data.

### 2.5. Assessment Measures

A sociodemographic questionnaire specifically developed for this research was administered, which included items on gender identity, age, and self-reported clinical diagnoses of mental disorders. Given the high diagnostic heterogeneity within the sample, participants were categorized into mutually exclusive groups based on self-reported clinical information and records from collaborating organizations. The final sample included five groups: (1) people without mental disorder, (2) psychotic disorder, (3) mood disorder, (4) substance use disorder, and (5) dual pathology, the latter being defined as the coexistence of a mental disorder and a substance use disorder.

In addition, the Adolescent/Adult Sensory Profile (AASP) [33], validated for the Spanish population [39], was employed. The AASP [33] is a 60-item self-report questionnaire divided into six sensory categories (taste/smell, movement, visual, touch, activity level, and auditory), designed to assess behavioral responses to sensory experiences. It identifies four sensory processing patterns based on Dunn’s Model (low registration, sensation seeking, sensory sensitivity, and sensory avoiding) [55]. Each item is rated on a 5-point Likert scale according to the frequency of the individual’s responses to everyday sensory experiences. For each of the four quadrants, total scores can be classified into five ranges, from much less than most people to much more than most people, indicating how an individual’s sensory processing compares to the normative population [33]. These scores were calculated looking at the standard deviation, following the original indications of the AASP tool: much less than most people (lower than −2 SD), less than most people (lower than −1 SD), similar to most people (between −1 and +1 SD), more than most people (higher than +1 SD) and much more than most people (higher than +2 SD).

Internal consistency (Cronbach’s α) values range from 0.66 to 0.70, which are comparable to those reported in the Spanish cultural adaptation study of the instrument, where coefficients ranged from 0.69 to 0.73 [39].

Finally, to accurately assess sensory processing in populations living under unstable or stressful conditions, such as homeless people, the AASP was carefully adapted in collaboration with the author responsible for its validation on Spanish adults. Specific items were modified to better capture the unique experiences and challenges associated with homelessness, ensuring the tool was relevant and meaningful for this population.

### 2.6. Data Analysis

A descriptive analysis of the participants’ sociodemographic, clinical and sensory variables was conducted, reporting measures of central tendency (mean or median) and dispersion (standard deviation or interquartile range) for quantitative variables, as well as absolute and relative frequencies for qualitative variables. The normality of the variables was assessed using the Kolmogorov–Smirnov test. The Student’s *t*-test for independent samples was applied to normally distributed data, whereas the Mann–Whitney U test was used for non-parametric comparisons, ensuring that each analysis employed the most appropriate statistical test based on the underlying distribution of the variables. Results were evaluated with a 95% confidence level and a statistical significance of *p* < 0.05, and statistical analyses were performed using IBM SPSS Statistics (version 27) (Armonk, NY, US).

### 2.7. Ethical Considerations

The Social Research Ethics Committee of Universidad Castilla la Mancha (UCLM) granted ethical approval for the study (code 65692-H5K0). Participant data confidentiality was rigorously maintained, and all participants received an information sheet detailing the study’s key aspects and objectives before providing written informed consent. All these procedures adhered to the ethical principles of the Declaration of Helsinki and the provisions of Organic Law 3/2018, of 5 December, on the Protection of Personal Data and Guarantee of Digital Rights.

## 3. Results

The results of this study are presented according to: (1) sociodemographic and clinical characteristics; (2) sensory reactivity outcomes; (3) a comparative analysis between the normative data obtained in Spain and the PEH sample; and (4) an analysis of differences in sensory processing between individuals with a mental disorder and those without one.

### 3.1. Sociodemographic and Clinical Characteristics of Participants

A total of 150 individuals participated in the study, with a mean age of 47.43 years (±10.94) and an age range from 18 to 73 years. Of the participants, 80.7% were men, 66.7% had a Spanish nationality, 62.7% were single and 71.3% reported having a mental health disorder. Table 1 presents the sociodemographic and clinical characteristics of the sample.

### 3.2. Sensory Reactivity Results of the Sample

The mean AASP score of 150 PEH was analysed to examine sensory processing patterns. Table 2 presents the results according to the quadrants of Dunn’s Sensory Processing Model (low registration, sensation seeking, sensory sensitivity, and sensation avoidance), as well as sensory factors (taste/smell, movement, visual, touch, activity level, and auditory) for the total sample and divided by gender (men and women).

Regarding the sensory quadrants, the highest mean score was obtained in sensation seeking (M = 49.97, SD = 10.29), while the lowest was found in low registration (M = 36.97, SD = 10.79). As for the sensory factors, the highest mean score was observed in the touch factor (M = 36.59, SD = 7.92), and the lowest in movement (M = 21.54, SD = 5.32). With respect to gender (men and women), no statistically significant differences were found in either sensory quadrants or sensory factors.

### 3.3. Comparative Analysis Between Normative Data from Spain and People Experiencing Homelessness

Table 3a,b presents the comparative analysis between the data obtained in the present study for PEH and the raw scores corresponding to the normative values for Spain from the AASP [56]. Data are presented according to the scores of the sensory quadrants (low registration, sensation seeking, sensory sensitivity, and sensation avoiding) and the typical ranges of sensory processing, divided by age groups (adults [18–64 years] and older adults [≥65 years]). In the adult group (18–64 years) significant differences were found in the quadrants of low registration (Z = 6.971, *p* < 0.001), sensory sensitivity (Z = 4.338, *p* < 0.001), and sensation avoiding (Z = 7.829, *p* < 0.001), indicating higher mean scores and typical ranges that were higher or much higher among PEH compared to the normative population (Table 3a). In the older adult group (≥65 years) statistically significant differences were observed in the sensation seeking quadrant (Z = 2.207, *p* = 0.027), indicating higher scores and typical ranges above the normative values which were classified as higher or much higher in the PEH group (Table 3b).

Table 4 presents the AASP data by sensory quadrants and factors among PEH, grouped according to self-reported mental disorder diagnostic. The Mann–Whitney U test was used to compare the sensory quadrant and factor scores between PEH with and without a self-reported mental disorder. No statistically significant differences were found for the sensory quadrants of low registration (Z = 0.260, *p* = 0.795), sensation seeking (Z = 0.329, *p* = 0.743), sensory sensitivity (Z = −0.110, *p* = 0.912), or sensation avoiding (Z = 0.586, *p* = 0.558). However, statistically significant differences were observed for the tactile sensory factor (Z = −2.529, *p* = 0.011) within the substance use disorder group (Me = 35.00, IQR = 13).

## 4. Discussion

The aim of this study was to analyse and describe the sensory processing patterns of PEH, as well as to determine whether their sensory reactivity differs from the normative population or if there are differences in PEH with and without mental disorders.

A total of 150 homeless people participated in our study. The sample analysed was predominantly male with a mean age of 47.43 years, consistent with data reported by the Spanish National Statistics Institute (INE) [3]. A lower incidence was observed among older adults (4%), possibly associated with the high mortality rates present in homeless people [57], largely resulting from chronic illnesses, mental health disorders, substance abuse and prolonged exposure [58,59], which are in turn affected by structural factors associated with homelessness in Spain, such as increasing housing costs, economic inequality, and job insecurity [60]. All of which contribute to premature mortality in PEH.

Regarding nationality, most participants were Spanish (66.7%), followed by people who migrated from Europe, Africa and South America. These findings differ from data reported by INE [3], where a more balanced distribution by nationality is shown. This discrepancy may reflect recent social and political changes in Spain, including the financial crisis, widespread evictions, the COVID-19 pandemic, rising housing costs and long-term unemployment [61,62].

In terms of marital status, single participants predominate, followed by the category of divorced. These results could be explained by social identity theory, which states that a loss of social status, derived by factors such as unemployment, or the disruption of traditional gender roles, can negatively impact self-esteem. Moreover, previous studies indicate that men tend to have smaller social support networks, which increases the risk of losing family ties [63].

Concerning the clinical variable of self-reported mental disorder diagnoses, 78% of participants reported having some form of mental disorder, a finding consistent with previous research that indicates this population is highly vulnerable to mental health problems [64,65], particularly anxiety, depression and substance use disorders [17,18].

With regard to the descriptive results on sensory processing patterns, clinical differences were found in the sample of PEH across all sensory quadrants of Dunn’s model [37]. Our findings show higher scores in all of the quadrants (low registration, sensation seeking, sensory sensitivity and sensation avoiding), with values exceeding the typical range of the normative population, reaching levels categorized as “more than most people” and “much more than most people” among PEH.

When comparing the results of PEH with the normative population for Spain [56], statistically significant differences in sensory processing were found. These findings indicate that PEH from the adult group aged 18 to 64 showed higher scores in low registration (Z = 6.971, *p* ≤ 0.00), sensory sensitivity (Z= 4.338, *p* ≤ 0.00), and sensation avoiding (Z = 7.829, *p* ≤ 0.00). While in the older adult group, with ages ranging from 65 years, statistically significant differences were observed in sensation seeking (Z = 2.207, *p* = 0.027), with higher scores in the PEH group.

In the present study, PEH exhibited clinically higher scores in low registration (indicating greater difficulties), which could be linked to the findings of Engel-Yegel, Gonda et al. [66], who suggested that individuals with low registration may present depressive mood states. Alternatively, Sanchis-Asensi [36] conducted a study with people diagnosed with schizophrenia in Spain, and reported that participants displayed a sensory profile characterized by low registration. These results reinforce the potential relevance and implications of analysing sensory processing patterns in PEH with mental health diagnoses.

Regarding sensation seeking, statistically significant differences were found in the sensation seeking quadrant among older adults (<65 years old), with higher scores in the PEH group. These characteristics may manifest as excessive displays of affection or attempts to initiate personal contact, which in some contexts may be perceived as risky or inappropriate behaviour [36]. The elevated clinical scores obtained in this quadrant among PEH (indicating greater difficulties) may be related to the onset of substance use problems, as was found in the study of Kelly et al. [67], conducted with 87 adults. These findings are consistent with those reported by LaBrie et al. [68], who identified sensation seeking as a predictor of alcohol consumption, and by Dervaux et al. [69], who found an association between sensation seeking and substance abuse among people with schizophrenia. The urge to consume substances may arise as a way of coping with stressful social events [70], social insecurity [71], or difficulties in social participation, as well as a form of self-medication for mental health problems and psychological distress [72,73,74].

In the case of sensory sensitivity, the results obtained in this study may be related to heightened hypervigilance in response to potential social threats commonly experienced by PEH, derived from characteristics such as sleeping on the streets. Furthermore, Van den Boogert et al. [23], conducted a systematic review that included 33 studies in which the AASP tool was employed, and it was found that hypervigilance has been widely associated with anxiety, a mental health condition frequently reported among PEH. It is also worth noting that authors like Halperin and Falk-Kessler [75] have reported impairments in the performance of activities of daily living among individuals with high sensory sensitivity. While Pfeiffer, Kinnealey, Reed and Herzberg [76] found that elevated sensory sensitivity scores were associated with reduced community participation, poorer quality of life and lower health recovery rates.

Concerning sensation avoiding, the high sensation avoidance scores observed in PEH may be related to the use of drugs or alcohol that is frequent in homelessness [67,77]. The consumption of these substances can alter sensory perception and tolerance, serving as coping mechanisms to reduce sensory overload and distance themselves from their living situation. Bashapoor et al. [78] found that people who have substance abuse problems engage in avoidance behaviours as a form of adaptation to social isolation and negative social interactions, which originate from a difficulty to identify emotions. Additionally, Neal et al. [79] described a relationship between behavioral inhibition and symptoms of anxiety and depression, particularly in social anxiety disorder. Both of these studies could explain the high scores obtained in PEH given the high prevalence of mental health disorders in the homeless population.

With regard to the presence of mental illness among PEH, no statistically significant differences were observed in sensory processing alterations between participants with a self-reported mental health diagnosis and those without. These results may be directly related to the sample size of each diagnostic subgroup. The number of participants in each group decreased substantially when stratifying the sample by this category, which could have rendered these subgroups as less representative of the overall sample. Another possible explanation for the absence of significant differences is the lack of variables that directly reflect the specific characteristics of the PEH population, such as social exclusion, isolation, limited opportunities for engaging in meaningful occupations and the stigma associated with homelessness [80,81]. These factors, which are intrinsically linked to the lived experience of homelessness, may exert a considerable influence on both sensory processing and mental health, and their omission from the analyses could, therefore, have limited the detection of significant associations.

However, a statistically significant relationship was found within the substance use disorder group in the tactile sensory factor, indicating that this group exhibited lower tactile processing compared to PEH with other mental health conditions (Z = −2.529, *p* = 0.011). These results may be related to reduced tactile sensitivity associated with paraesthesia, numbness or hypoesthesia, frequently experienced by PEH who present a prolonged and heavy alcohol consumption [82,83]. Moreover, additional factors such as malnutrition, chronic exposure to cold environments and poor hygiene may increase the risk of peripheral neuropathies, further aggravating these symptoms and compromising the quality of life of this population [84,85].

These findings suggest that PEH present sensory profiles that differ from those of the normative population. PEH interact within unpredictable and unstable environments characterized by a high level of stress-inducing stimuli such as auditory (noise), olfactory (smells) and visual (bright lights) factors among others, over which they have little to no control. Daily activities are often repetitive and lack purpose, leading to social isolation and a reduction in meaningful engagement. The absence of support networks and the challenges in establishing effective relationships with professionals within homeless service systems may further contribute to these sensory and behavioral differences when compared to the normative population.

Our findings extend existing knowledge by applying Dunn’s model to consider environmental determinants and the social context, an area that had not been previously explored. This novel approach enables the examination of sensory processing dimensions beyond traditional clinical settings, which provides preliminary evidence of their relevance for understanding the everyday experiences of PEH.

Previous studies that have evaluated sensory profile characteristics using the AASP tool have highlighted the importance of addressing sensory processing difficulties, as these are associated with reduced participation and enjoyment in activities [31,86], and the appearance of challenges that affect daily life [23]. Interventions based on the analysis of sensory profiles can offer a non-invasive approach that provides both individuals and professionals a more comprehensive understanding of how to manage environmental demands. Such interventions can be tailored to meet individual needs by regulating sensory input and creating safe and supportive environments that promote recovery and address issues related to homelessness. In particular, occupational therapists are able to design these types of environments in order to adapt individual sensory preferences and foster self-regulation strategies. These strategies introduce an innovative perspective for creating personalized interventions that are sensitive to individual needs and focused on reducing vulnerability associated with social exclusion [87], granting an approach that can be applied in social contexts aimed at enhancing participation [36]. This model advocates five key components of intervention: (1) education and understanding, (2) self-advocacy, (3) sensory diet, (4) environmental adaptation and (5) social support [88].

The practical implications of this approach could be directed toward creating more comfortable and supportive environments for interventions with this population. Shelters for PEH often display a neglected or abandoned appearance, which may convey a sense of hopelessness and reinforce resignation to the situation of homelessness. Many shelters for homeless people become hostile rather than calm and harmonious places, and they frequently lack outdoor areas that promote social interaction and facilitate group activities.

Some recommendations for interventions with this population could include providing spaces that help regulate sensory stimuli, taking into account factors such as auditory (soundproof rooms, use of headphones or earplugs, volume control), visual (adjustable lighting, use of eye masks), olfactory (air fresheners or purifiers, plants, windows that open), tactile (temperature control, availability of varied textures in household items such as blankets or towels, mats in rest areas), gustatory (access to condiments such as spices or salt), movement (outdoor spaces for physical activity) and activity level (areas that facilitate engagement in daily or group activities) [89,90]. Taking this into account, support could be provided to enhance occupational performance and self-regulation strategies aimed at identifying stressful sensory inputs and developing adaptive responses to them.

In conclusion, an approach based on sensory profiling would not only improve the overall well-being and outlook of PEH, but could also enhance adherence to recovery programs and increase participation in meaningful occupations.

### 4.1. Limitations

When interpreting the results of this study, several limitations should be taken into consideration. First, this was an observational, descriptive, and cross-sectional study with a relatively small sample size, particularly when the sample was divided according to self-reported mental health diagnoses. Therefore, caution should be exercised when interpreting the results and attempting to generalize them, as they may not be fully representative of the broader population.

Secondly, the sample was obtained through convenience sampling due to the challenges associated with accessing and locating individuals experiencing homelessness. Although this sampling method limits the generalization of the results, in exploratory studies such as this one, it is often considered useful as it allows for the collection of valuable firsthand information.

Thirdly, the clinical diagnosis variable was obtained from self-reported information provided by participants and was not verified by a medical professional, which could imply that the data collected presents a certain degree of inaccuracy. Fourthly, the gender distribution of the sample was uneven, with a marked overrepresentation of men compared to women. Although this pattern aligns with previous research on populations experiencing homelessness, future studies should consider sampling strategies that promote a more balanced gender representation.

Finally, some variables related to individual experiences of PEH were not included, and the evaluation of certain areas could not be made as there are no standardized tools available for this population. Characteristics such as duration of homelessness or educational level may be associated with coping strategies and sensory processing patterns and were not included in the analysis.

### 4.2. Future Research

Given that this was the first study to examine the relationship between PEH and sensory processing, future research should further examine this association and take into account clinical variables such as the presence of mental disorders specifically diagnosed by medical professionals.

Likewise, it would be relevant to investigate how the living conditions of this collective, such as exposure to stressful urban environments or residence in shelters or day centers, may relate to sensory processing patterns. Another potential line of research involves the development of sensory-based interventions that implement self-regulation strategies, assessing their impact on the functionality and social adaptation of PEH through longitudinal or qualitative studies.

## 5. Conclusions

Sensory processing has not previously been examined in people experiencing homelessness, and our findings indicate that it represents a relevant domain to consider in this highly vulnerable population. PEH showed significant differences in sensory processing, specifically in low registration, sensory sensitivity, and sensation avoiding among adults aged 18 to 64 years, and in sensation seeking among those aged 65 years and older, compared to individuals with typical development. In addition, among the sensory systems, only tactile processing showed a reduction in responsiveness of stimuli among those with a diagnosis of substance use disorder. These results suggest that sensory processing can influence how PSH perceive and respond to their environment.

As previously mentioned, this was the first study to apply Dunn’s model within environmental and social context of homelessness, offering initial evidence of sensory processing dimensions that are relevant for understanding the everyday experiences of PEH. Incorporating the evaluation of the Adolescent/Adult Sensory Profile (AASP) into PEH could facilitate the design of personalized care. Therefore, it is essential to assess each person’s sensory profile and explicitly consider how to support their sensory needs, in order to promote a holistic approach.

## Figures and Tables

**Table 1:** Sociodemographic and clinical characteristics of the sample (*n* = 150).

		n	%
Gender	Men	121	80.7
	Women	29	19.3
Age	18–64 years old	144	96.0
	65+ years old	6	4.0
Nationality	Spain	100	66.7
	Europe	17	11.33
	Africa	19	12.67
	South-America	14	9.33
Civil status	Single	94	62.7
	Married	8	5.3
	Legally separated/divorced	38	25.3
	Separated arranging legal proceedings	4	2.7
	Widowed	5	3.3
	Other	1	0.7
Self-reported mental illness diagnosis	Without mental disorder	43	28.7
	Psychotic disorder	13	8.7
	Mood disorder	14	9.3
	Substance use disorder	35	23.3
	Dual pathology	45	30.0

**Table 2:** General characteristics of PEH (*n* = 150) by quadrants and sensory factors, with gender-based differences.

	Total Sample		Gender					
	PEH (n = 150)		Men (n = 121)		Women (n = 29)		Student’s *t*-Test	
	M	SD	M	SD	M	SD	t	*p*
Low registration	36.97	10.79	37.20	10.38	36.03	12.49	1.268	0.262
Sensation seeking	49.97	10.29	49.96	10.18	50.00	10.91	0.001	0.977
Sensory sensitivity	41.36	10.36	40.89	40.00	43.31	10.21	0.047	0.828
Sensation avoiding	45.61	12.07	44.64	12.17	49.66	10.92	1.817	0.180
Taste/smell	23.97	5.43	23.86	5.42	24.41	5.52	0.019	0.892
Movement	21.54	5.32	21.48	5.13	21.79	6.17	2.817	0.095
Visual	29.50	7.69	29.37	7.84	30.03	7.12	0.759	0.385
Touch	36.59	7.92	36.03	7.99	38.93	7.28	0.224	0.637
Activity level	29.83	6.11	29.91	6.10	29.52	6.24	0.000	0.995
Auditory	32.35	8.08	31.88	8.07	34.31	7.96	0.001	0.970

Note: The Student’s *t*-test was performed to compare men and women. M = mean, SD = standard deviation. A 95% confidence level was applied in all statistical analyses.

**Table 3:** (**a**) Comparative analysis of normative values and the study sample of PEH, grouped by aged 18–64. (**b**) Comparative analysis of normative values and the study sample of PEH, grouped by aged < 65.

Sensory Quadrants	(a) Aged 18–64								
	Normative Values			Homeless People			Comparison		
	M	SD	Typical Range	M	SD	Typical Range	t	*p*	d
Low registration	29.26	6.56	23–37	36.95	10.88	26–48	8.486	<0.001 **	0.44
Sensation seeking	48.63	7.50	41–56	49.80	10.43	39–60	1.344	0.181	0.06
Sensory sensitivity	37.08	8.71	28–46	41.13	10.92	30–52	4.453	<0.001 **	0.20
Sensation avoiding	35.38	7.59	28–43	45.57	12.10	33–58	10.104	<0.001 **	0.52
**(b) Aged < 65**									
Low registration	34.86	8.74	26–44	37.50	9.12	28–47	2.307	0.510	0.15
Sensation seeking	43.31	9.28	34–53	54.00	10.69	43–65	7.463	<0.001 **	0.53
Sensory sensitivity	42.07	10.05	32–52	37.83	6.11	32–44	−1.031	0.150	0.25
Sensation avoiding	41.94	9.52	32–51	46.67	12.29	34–59	3.599	0.389	0.22

Note: M = mean, SD = standard deviation, *p* = probability value, t = t statistic, d = Cohen’s d. *p* values were obtained using the paired-samples Student’s *t*-test. The typical ranges reported for PEH were generated based on the mean scores of the PEH sample in the present study (n = 150). Reference values and typical ranges for the normative data were extracted from Gándara-Gafo, Santos-del Riego et al. [56]. A 95% confidence level was applied in all statistical analyses. ** High statistical significance.

**Table 4:** Sensory quadrants and sensory factors in PEH, according to self-reported mental disorder diagnostic groups.

Self−Reported Mental Disorder Diagnosis															
	People Without Mental Disorder (n = 43)			Psychotic Disorder (n = 13)			Mood Disorder (n = 14)			Substance Use Disorder (n = 35)			Dual Pathology (n = 45)		
	Me (IQR)	Z	*p*	Me (IQR)	Z	*p*	Me (IQR)	Z	*p*	Me (IQR)	Z	*p*	Me (IQR)	Z	*p*
Low Registration	35.00 (17)	−0.260	0.795	31.00 (23)	−0.956	0.339	40.50 (14)	0.976	0.329	33.00 (12)	−0.534	0.593	39.00 (15)	1.092	0.275
Sensation seeking	50.00 (13)	0.329	0.743	51.00 (14)	0.468	0.640	49.50 (15)	−0.145	0.884	52.00 (17)	−1.176	0.240	49.00 (14)	−1.104	0.270
Sensory sensitivity	41.00 (15)	−0.110	0.912	39.00 (16)	−0.190	0.849	43.50 (18)	0.614	0.539	42.00 (18)	−0.790	0.430	40.00 (16)	.074	0.941
Sensation avoiding	46.00 (21)	−0.586	0.558	48.00 (17)	1.353	0.176	47.00 (22)	−0.123	0.902	50.00 (20)	−0.260	0.795	46.00 (20)	−1.048	0.294
Taste/smell	26.00 (9)	1.029	0.304	22.00 (8)	−1.881	0.060	23.00 (4)	−0.210	0.833	25.00 (8)	0.470	0.639	24.00 (8)	−0.031	0.975
Movement	20.00 (8)	−0.546	0.585	19.00 (12)	−0.315	0.753	22.00 (9)	0.340	0.734	20.00 (6)	0.521	0.603	21.00 (5)	0.835	0.404
Visual	30.00 (14)	−0.766	0.444	33.00 (8)	1.026	0.305	28.00 (16)	−0.262	0.793	30.00 (11)	−0.964	0.335	31.00 (9)	0.511	0.609
Touch	37.00 (14)	0.250	0.803	38.00 (12)	0.321	0.748	34.50 (11)	0.437	0.662	35.00 (13)	−2.529	0.011 *	37.00 (10)	−0.447	0.655
Activity level	28.00 (13)	−1.186	0.236	29.00 (12)	0.682	0.495	31.50 (9)	1.064	0.287	30.00 (10)	−0.224	0.823	29.00 (6)	−0.815	0.415
Auditory	32.00 (10)	−0.073	0.942	36.00 (20)	0.572	0.568	35.50 (13)	0.575	0.565	31.00 (10)	−1.457	0.145	33.00 (15)	0.045	0.964

Note: The Mann–Whitney U test was performed to compare PEH with and without a self-reported mental disorder. Me = median, IQR = interquartile range. Z = Z statistic from the Mann–Whitney U test; *p* = *p*-value (statistical significance). A 95% confidence level was applied in all statistical analyses. * Statistically significant.

## Data Availability

The datasets analysed during the current study are not publicly available in order to protect the privacy and confidentiality of the participants, but are available from the corresponding author upon reasonable request.

## Abbreviations

The following abbreviations are used in this manuscript:

PEH	People experiencing homelessness
FEANTSA	European Federation of National Organisations Working with the Homeless
ETHOS	European Typology on Homelessness and Housing Exclusion
AASP	Adolescent/Adult Sensory Profile
STROBE	Strengthening the Reporting of Observational studies in Epidemiology

## Appendix A

### European Typology of Homelessness and Housing Exclusion

**Table A1:** Main categories of the ETHOS scale.

	Operational Category		Living Situation		Generic Definition
ROOFLESS	1	People Living Rough	1.1	Public space or external space	Living in the streets or public spaces, without a shelter that can be defined as living quarters
	2	People in emergency accommodation	2.1	Night shelter	People with no usual place of residence who make use of overnight shelter, low threshold shelter
HOUSELESS	3	People in accommodation for the homeless	3.1	Homeless hostel	Where the period of stay is intended to be short term
			3.2	Temporary accommodation	
			3.3	Transitional supported accommodation	
	4	People in Women’s Shelter	4.1	Women’s shelter accommodation	Women accommodated to experience of domestic violence and where the period of stay is intended to be short term
	5	People in accommodation for immigrants	5.1	Temporary accommodation/reception centers	Immigrants in reception or short-term accommodation due to their immigrant status
			5.2	Migrant workers accommodation	
	6	People due to be released from institutions	6.1	Penal institutions	No housing available prior to release
			6.2	Medical institutions (*)	Stay longer tan needed due to lack of housing
			6.3	Children’s institutions/homes	No housing identified (e.g., by 18th birthday)
INSECURE	7	People receiving longer-term support (due to homelessness)	7.1	Residential care for older homeless people	Long stay accommodation with care for formerly homeless people (normally more than one year)
			7.2	Supported accommodation for formerly homeless people	
	8	People living in insecure accommodation	8.1	Temporarily with family/friends	Living in conventional housing but not the usual place of residence due to lack of housing
			8.2	No legal (sub)tenancy	Occupation of dwelling with no legal tenancy illegal occupation of a dwelling
			8.3	Illegal occupation of land	Occupation of land with no legal rights
	9	People living under threat of eviction	9.1	Legal orders enforced (rented)	Where orders of eviction are operative
			9.2	Re-possession orders (owned)	Where mortgagee has legal order to re-possess
	10	People living under threat of violence	10.1	Police recorded incidents	Where police action is taken to ensure place of safety for victims of domestic violence
INADEQUATE	11	People living in temporary/non-conventional structures	11.1	Mobile homes	Not intended as place of usual residence
			11.2	Non-conventional building	Makeshift shelter, shack or shanty
			11.3	Temporary structure	Semi-permanent structure hut or cabin
	12	People living in unfit housing	12.1	Occupied dwellings unfit for habitation	Defined as unfit for habitation by national legislation or building regulations
	13	People living in extreme over-crowding	13.1	Highest national norm of overcrowding	Defined as exceeding national density standard for floor-space or useable rooms

Note: This table was adapted from the European Typology of Homelessness and Housing Exclusion [4]. (*) Includes drug rehabilitation institutions, psychiatric hospitals, etc.
